# Simulation of the Frank-Starling Law of the Heart

**DOI:** 10.1155/2012/267834

**Published:** 2012-11-29

**Authors:** Samo Ribarič, Marjan Kordaš

**Affiliations:** Institute of Pathophysiology, Faculty of Medicine, University of Ljubljana, 1104 Ljubljana, Slovenia

## Abstract

We developed a lumped parameter, computer-based model of an equivalent electronic circuit for a one-atrium one-ventricle (frog) heart attached to a vascular circuit, to simulate a basic concept of cardiovascular physiology, the Frank-Starling Law of the Heart. A series of simulations was performed, to observe changes in cardiovascular variables (e.g., arterial pressure, ventricular volume, and valve flows) if either preload or afterload was increased. The simulated data agreed qualitatively, and quantitatively when experimental data are available, with data obtained on amphibian or on mammalian myocardium. In addition, the data obtained in these simulations improve our understanding of the mechanism(s) whereby the heart muscle adapts itself to increased distension (increased preload) or to impeded systolic contraction (increased afterload). The analysis of the measured valve flows suggests that the ventricle is a highly input sensitive pump because the input pressure determines the diastolic distension and, consequently, the force of ventricular systolic contraction. On the other hand, the ventricle is a relatively output insensitive pump. Therefore, not only atrium contraction, but also predominantly the preceding ventricular systolic contraction is the main mechanism of the subsequent diastolic ventricular filling. We conclude that the presented model enables the study of basic concepts of cardiovascular physiology.

## 1. Introduction

Originally, cardiovascular physiology was simulated by mathematical [1–4] or analog [5–8] approaches. Recently, computer simulations using lumped parameter models of the cardiovascular system (alone or in combination with numerical methods) have been introduced [9–15]. We have found the use of lumped parameter models with computer-simulated equivalent electronic circuits (EECs) useful for understanding basic physiology concepts and for educational use [16, 17]. By appropriate upgrading, we could use these EECs for simulating qualitatively, in some cases even quantitatively, cardiovascular physiology and cardiovascular pathology, including well-known clinical conditions [18, 19]. This highly upgraded EECs lumped parameter model enables us to record many interesting variables, for example, the time course of atrial and ventricular pressure during systole and diastole, the rate of myocardial contraction and relaxation, the valve flows, the ventricular volume taking into consideration the resetting of the sympathetic nervous tone in the heart and systemic circuit, the fluctuating intrathoracic pressure during respiration, and the passive relaxation of ventricle during diastole.

In clinical practice, and in whole animal or human studies, the short-term and long-term compensatory mechanisms modify cardiac function thus impeding the study of the basic mechanical properties of the whole heart in isolation. Therefore, it would be of interest to study, by using the EECs model, the basic determinants of myocardial function(s) as it was originally attempted by using the *in vitro* frog heart model [20, 21]. It should be remembered that it is a one-atrium one-ventricle preparation. Its function, determined by atrial pressure (input pressure, preload) and by output pressure (afterload), is formulated as the Frank-Starling Law of the Heart [20].

In the presented simulation of the Law of the Heart it is attempted to answer the following basic questions.

1. *What is the role of atrium in the filling of the ventricle, if the latter is subjected to increased preload or increased afterload?*
2. *Why is the ventricle a highly input sensitive pump, but at the same time a relatively output insensitive pump?*
3. *What is the change in the pressure-volume (P-V) loop diagram if the ventricle is subjected to increased preload or increased afterload?*

## 2. Methods

**Figure 1:**
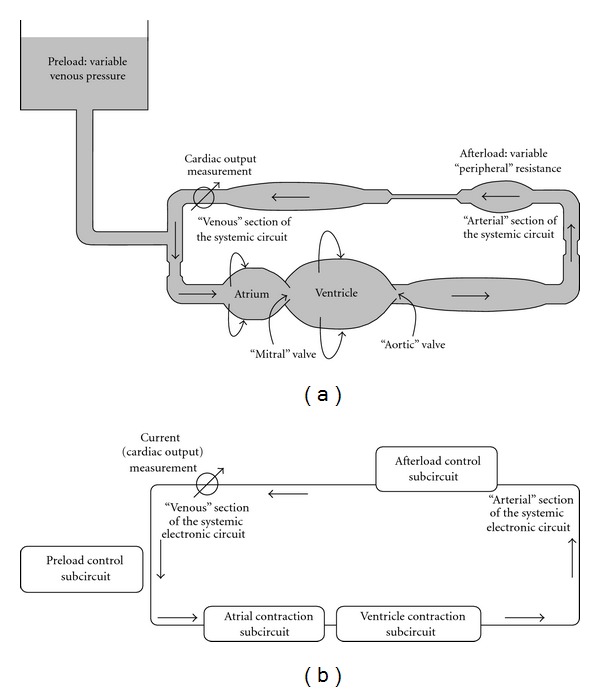
Representation of the one-atrium one-ventricle heart preparation. (a) Schematic representation of a one-atrium one-ventricle heart, the contractility of both being determined by their diastolic distension. If it is contracting, flow is unidirectional due to both valves, the “mitral” and “aortic” valves. This one-atrium one-ventricle heart is attached to an artificial circulation, set up by three sections: (1) an elastic “arterial” high-pressure section, (2) a “peripheral,” variable resistance section, and (3) a “venous” low-pressure section. (b) Schematic representation of the EEC simulating the properties of a one-atrium one-ventricle heart attached to an artificial circulation. Current (equivalent to blood flow) is measured at the end of the “venous” section because at this point of the circuit pulsations of the current are minimal.

In the experimental layout the one atrium-one ventricle heart (e.g., of the frog) should be attached to an artificial “systemic” circuit, featuring three characteristic sections: (a) a high-pressure/low-capacitance “arterial” section, (b) a variable “peripheral” resistance section, and (c) a low-pressure/high-capacitance “venous” section including a blood reservoir. In this circular arrangement pressures, flow, and stroke volume would depend on preload and afterload. Preload is determined by the height of venous reservoir, and afterload is controlled by “resistance to flow” section (Figure 1(a)). If the heart is contracting, flow is unidirectional due to valves, the “input” and “output” valve. In the “arterial,” high-pressure section, pressure depends predominantly on afterload, that is, on the variable resistance to flow and on the cardiac output. Contrary to that, in the “venous” low-pressure section, pressure depends predominantly on preload, that is, the height of blood level in the venous reservoir.

The equivalent electronic circuit (EEC) should have a very similar structure; the “systemic” circuit, preload control subcircuit, afterload control subcircuit, atrial contraction subcircuit, and ventricular contraction subcircuit, as shown in Figure 1(b). Parallel to the scheme in Figure 1(a), electronic subcircuits simulate preload, atrial contraction, ventricular contraction, and afterload. These subcircuits are suitably incorporated into the main circuit simulating the “arterial” section, the “peripheral resistance section,” and “venous” section of systemic circuit.

It should be noted that, for reasons of comparison, in the present EEC both atrium and ventricle are comparable to the adult human left atrium and left ventricle. Therefore, in the latter, in resting pressure and capacitance conditions, its volume (size, EDVV) is about 117 mL and peak “arterial” pressure is about 70 mm Hg. For reasons of simplicity, in simulations the usual terminology is used also for both valves in this one-atrium one-ventricle heart: at the input of the ventricle the mitral and at the output the aortic valve, respectively. 

Analysis of the EEC simulating the one-atrium one-ventricle heart, attached to an artificial vascular circuit, is performed by using Electronics Workbench Personal version 5.12 [22]. This software is used because the circuitry can be tailored almost exactly to the simulation needs. The conversion of electrical units to equivalent units used in physiology is shown in Table 1.

**Figure 2:**
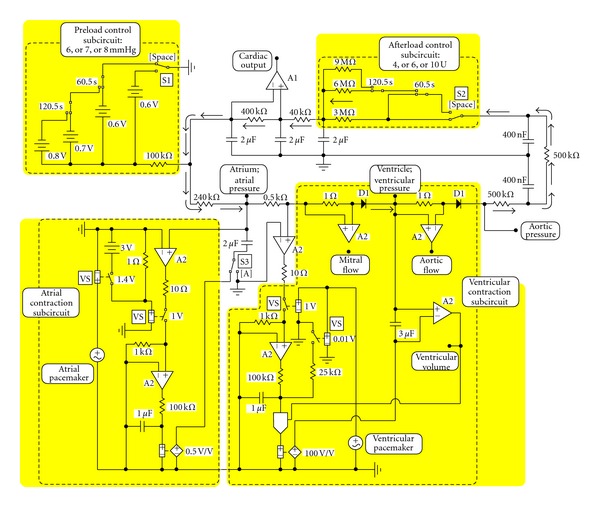
Electronic circuit simulating the one-atrium one-ventricle heart and the artificial systemic circuit. Subcircuits (for explanation see Figure 1(b)) are indicated by dashed-lined and shaded boxes. The mode of simulation is determined by 3 switches: S1 and S2 operated by the “space” key, and S3 operated by the “A” key. Various variables can be recorded and measured at points as indicated: pressures (e.g., atrial, ventricular, and “aortic”); flows (e.g., mitral, aortic, and cardiac output); volumes (e.g., ventricular). The “systemic” circuit is made up by a chain of resistor/capacitor segments. The direction of flow (current) is indicated by arrows.

The EEC simulating the one-atrium one-ventricle heart and the artificial systemic circuit is shown in Figure 2. The components (as described in Figures 1(a) and 1(b)) are simulated by subcircuits and indicated by dashed-lined boxes.

Details of how to simulate the atrium and ventricle—both as input-sensitive pumps—and the attached vessels are described elsewhere (e.g., [17–19, 23]). To summarize, the main sections of the EECs model are as follows.

**Figure 3:**
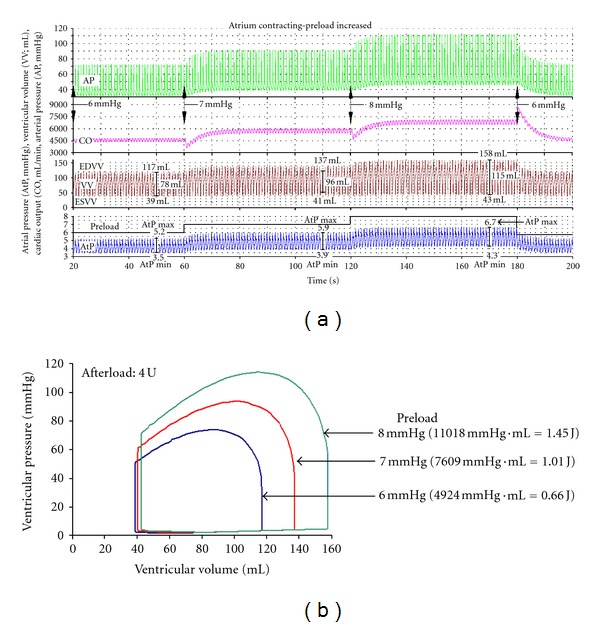
The time course of aortic pressure (AP), cardiac output (CO), ventricular volume (VV), and atrial pressure (AtP) if afterload is constant (4 U) and preload increased from 6 mm Hg to 8 mm Hg. (a) In initial conditions (20–60 s; preload is 6 mm Hg) all variables are in steady state. Atrial pulse pressure (AtP max/AtP min) is 5.2/3.5 mm Hg. Preload increased first to 7 mm Hg (60–120 s) and then to 8 mm Hg (120–180 s). Note that at each step CO is transiently decreased, but within about 10 s it is increased to a new steady-state level. Similarly, SV and AP are increased. AtP pulse is increased. After preload is returned to 6 mm Hg, AP, AtP, and VV return to initial conditions level. CO is transiently increased but within about 15 s it also returns to the initial condition level. (b) The P-V loop diagram in conditions above. Note that, if preload is increased from 6 mm Hg to 8 mm Hg, ESVV is about 40 mL, while EDVV is increased from 117 mL to about 158 mL. The EF increases from 66% to about 72%. Ventricular work is given in mm Hg·mL and in J.

**Figure 4:**
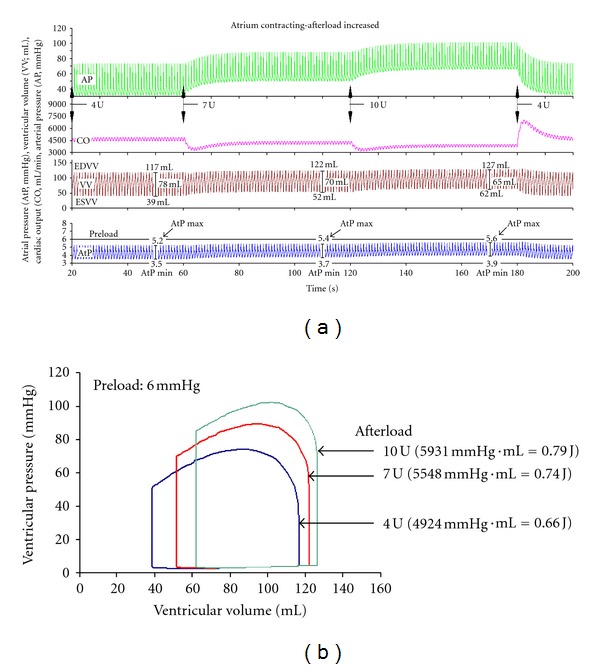
The time course of aortic pressure (AP), cardiac output (CO), ventricular volume (VV), and atrial pressure (AtP) if preload is constant (6 mm Hg) and afterload increased from 4 U to 10 U. (a) In initial conditions (20–60 s; afterload is 4 U) all variables are in steady state. Atrial pulse pressure (AtP max/AtP min) is 5.2/3.5 mm Hg. Afterload increased first to 4 U (60–120 s) and then to 10 U (120–180 s). Note that at each step CO is transiently significantly decreased, but within about 20 s it recovers to a new steady state, slightly lower level. Similarly, SV is slightly decreased. AP is increased. AtP max and AtP min are increased to a different extent; therefore, AtP pulse pressure is decreased. After afterload is returned to 4 U, AP, AtP, and VV return to initial conditions level. CO is transiently increased but within about 15 s it also returns to the initial condition level. (b) The P-V loop diagram in conditions above. Note that, if afterload is increased from 4 U to 10 U, ESVV is increased from 39 mL to about 62 mL, while EDVV is increased to a smaller extent, from 117 mL to 127 mL. The EF decreases from 66% to 51%. Ventricular work is given in mm Hg·mL and in J.

**Figure 5:**
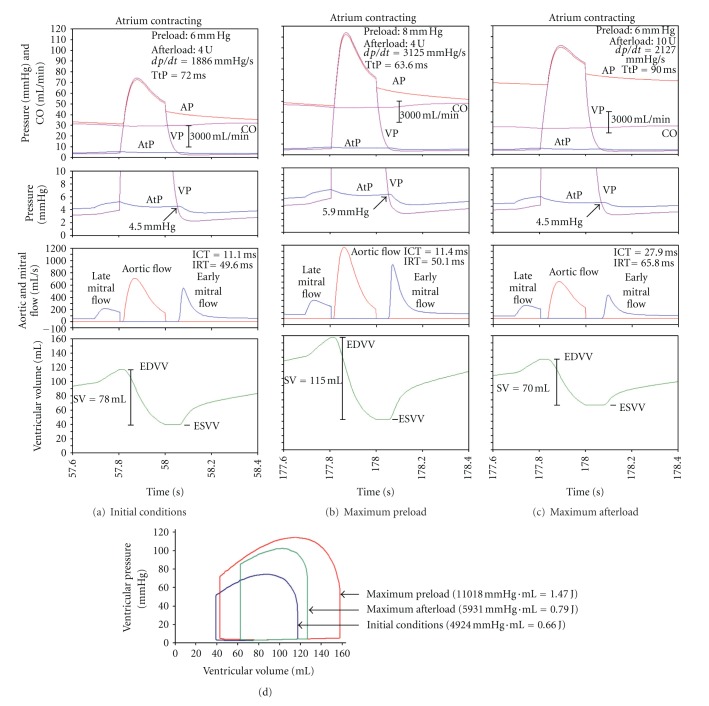
Sample values of cardiovascular variables in a single cardiac cycle during initial conditions (column a), maximal preload (column b), or maximal afterload (column c) when atrium *is contracting*. Each column is subdivided in four blocks. Upper two blocks: AP, VP, AtP, and CO (VP and AtP also at an enlarged pressure scale). Middle block: mitral flow and aortic flow. Bottom block: EDVV, ESVV, and SV. Note the “hump” in AtP in late diastole. Note also that the early mitral flow commences when AtP = VP. The higher is this pressure, the higher is peak early mitral flow. (a) Initial conditions (57.6 s–58.4 s of simulation time). Note that atrial contraction (immediately prior to the ventricular contraction) results in a late mitral flow, increasing EDVV by about 20 mL. Aortic flow is synchronous with ventricular contraction; this flow is stopped abruptly as soon as VP is lower than AP. Early mitral flow starts as soon as VP is lower than AtP. (b) Preload increased to 8 mm Hg (177.6 s–178.4 s of simulation time). Note that AP and VP are strongly increased. Similarly, both mitral flows and aortic flow are increased, and consequently EDVV, SV, and CO are strongly increased. Almost no change in ESVV, in ICT, and in IRT. dp/dt is increased from the resting value 1886 mm Hg/s to 3125 mm Hg/s; consequently, TtP is decreased from its resting value 72 ms to 63.6 ms. (c) Afterload increased to 10 U (177.6 s–178.4 s of simulation time). Note that both AP and VP are strongly increased. However, both mitral flows and aortic flow are decreased. Consequently EDVV and ESVV increase in an unequal extent, so that SV and consequently CO are slightly decreased. Note also that both ICT and IRT are increased. dp/dt is slightly increased from the resting value 1886 mm Hg/s to 2127 mm Hg/s; consequently TtP is increased from its resting value 72 ms to 90 ms. (d) The P-V loop diagram of the conditions above. If preload is increased, the loop diagram becomes broader. Both ventricular pressure and SV are increased. If afterload is increased, the loop diagram becomes narrower. Ventricular pressure is increased, but SV is slightly decreased. Ventricular work is given in mm Hg·mL and in J.

**Figure 6:**
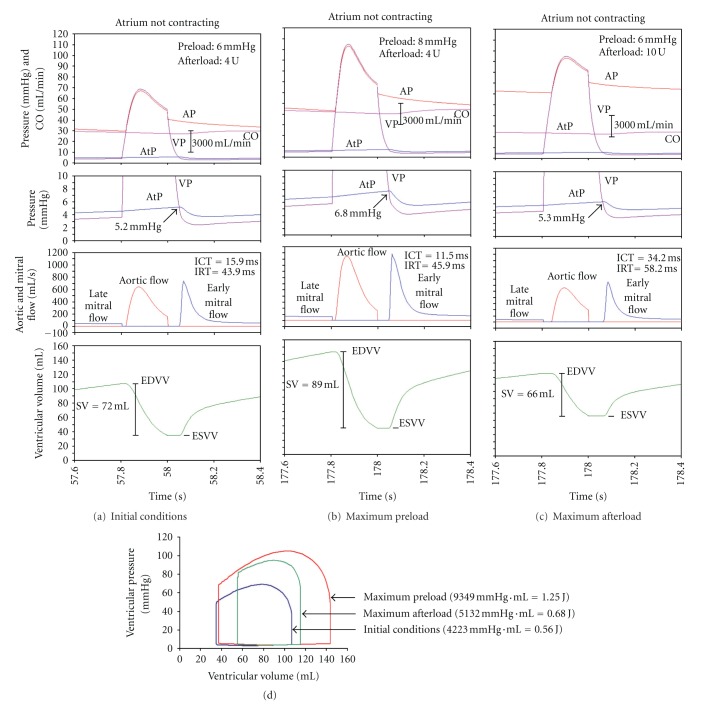
Sample values of cardiovascular variables in a single cardiac cycle during initial conditions (column a), maximal preload (column b), or maximal afterload (column c) when atrium *is not contracting*. Each column is subdivided in four blocks. Details are the same as in Figure 5. If atrium is not contracting, there is no “hump” in AtP in late diastole. However, also in this condition the early mitral flow commences when AtP = VP. However, AtP is higher (cf. Figure 5). Therefore, if atrium is not contracting, peak early mitral flow is higher. Data, presented in columns (a, b, and c), are qualitatively similar to those shown in Figure 5. Similarly, the P-V loop diagram (d) is qualitatively similar to that shown in Figure 5.

- The “systemic” circuit is made up by a chain of resistor/capacitor segments. The direction of flow (current) is indicated by arrows. Resistors are connected in series. One terminal of a single capacitor is connected to the chain of resistors and the other capacitor terminal is connected to ground. Thus, the resistor/capacitor segments simulate “resistance to flow” and capacitance of single segments of the circuit attached to the heart.
- Preload control subcircuit: by applying the 0.6 V, 0.7 V, and 0.8 V batteries (in parallel to the “systemic” circuit) preload can be set as 6 mm Hg, 7 mm Hg, or 8 mm Hg.
- Afterload control subcircuit: by applying the 3 MΩ, 6 MΩ, and 9 MΩ resistors (in series into the “systemic” circuit) afterload can be increased. Because the series resistance of aorta is 1 U (1 MΩ) afterload can be set as 4 U, or 7 U, or 10 U.
- Atrial contraction subcircuit: essentially, it is a feedback loop with a nominal gain of 0.5 and a nominal time constant 0.1 s. It is activated/deactivated by the atrial pacemaker as described below.
- Ventricle contraction subcircuit: it is a complex feedback loop with a nominal gain of 100. Nominal time constant of ventricular contraction is 0.1 s and that of ventricular relaxation is 0.2 s. Duration of systole is 0.2 s because this value has been used in almost all previous simulations. The gain loop is activated/deactivated by the ventricular pacemaker (synchronous—but with a time shift as described above—with the atrial pacemaker) and two voltage-sensitive switches VS.
- Heart valves: the mitral and aortic valves are simulated as diodes (D1; cf. [18, 19]). The corresponding mitral and aortic flow is measured as a voltage drop across the 1 Ω resistor (Figure 2) as described [18, 19].
- Design of the pacemaker circuit: it consists of two parts: an atrial and a ventricular pacemaker. The atrial pacemaker is a sine-wave generator operating at 1 Hz, (60/min), but with a phase shift of 192°, driving two voltage-sensitive switches (VS). The ventricular pacemaker is a square-wave generator operating at 1 Hz (60/min, duty cycle 0.2 s) which drives two voltage-sensitive switches (VS). In this way both generators, although physically separated, act as a single pacemaker, atrial contraction (nominal gain 0.5) preceding the ventricular contraction (nominal gain 100). The time course of simulated variables (e.g., ventricular pressure, volume of the ventricle; see Figures 3–6) is very similar to those described earlier [16].
- Possible modes of simulation: the mode of simulation is determined by 3 switches: S1 and S2 operated by the “space” key, and S3 operated by the “A” key.

In the setting presented in Figure 2 atrium is contracting. Afterload is constant, 4 U (3 MΩ + 1 MΩ), and preload is stepwise increased: initially 6 mm Hg (0.6 V). At 60.5 s it is increased to 7 mm Hg (0.7 V) and at 120.5 s of simulation time increased to 8 mm Hg (0.8 V). If the “space” key is operated, preload is constant, 6 mm Hg (0.6 V), and afterload stepwise increased: initially 4 U (3 MΩ + 1 MΩ). At 60.5 s it is increased to 7 U (6 MΩ + 1 MΩ) and at 120.5 s of simulation time increased to 10 U (9 MΩ + 1 MΩ). If the “A” key is operated, the capacitor simulating atrium is disconnected from its contractility circuit and connected to ground. Atrium is not contracting.

Variables of interest can be recorded and measured at points as indicated (Figure 2): pressures (e.g., atrial, ventricular, and “aortic”); flows (e.g., mitral, aortic, and cardiac output); volumes (e.g., ventricular).

The acronyms used in text and illustrations are listed in Table 2.

Data are shown as the time course of different variables and as P-V loop diagrams of the ventricle. The work load of the ventricle is calculated in the usual arbitrary units (mm Hg·mL) as well as in joule (J).

## 3. Results

### 3.1. EEC Simulation of the Law of the Heart with Contracting Atrium

The effects of increasing preload on the time course of AP, CO, and AtP are shown in Figure 3. In initial conditions (20–60 s of simulation time) preload is 6 mm Hg and afterload is 4 U. When, at 60 s, preload is increased to 7 mm Hg, AP, AtP, and SV are increased (Figure 3(a)). Note also that both AtP max and AtP min are increased; AtP pulse pressure is increased. CO is transiently decreased, but within about 10 s it is increased from about 4500 mL/min to almost 6000 mL/min. At the next step, at 120 s, as preload is increased from 7 mm Hg to 8 mm Hg, a similar further increase of these variables occurs. At 180 s, when preload returns to initial conditions, AP, AtP, and VV return to initial conditions level. CO is transiently increased but within about 15 s it also returns to the initial condition level. The performance (i.e., work load) of the ventricle in these simulation conditions is shown by the corresponding P-V loop diagram (Figure 3(b)). If preload is increased, the ESVV is only slightly increased. Contrary to that, EDVV and SV are strongly increased (cf. Figure 3(a)).

The effects of increasing afterload on the time course of AP, CO, and AtP are shown in Figure 4. In initial conditions (20–60 s of simulation time) preload is 6 mm Hg and afterload is 4 U. When, at 60 s, afterload is increased from 4 U to 7 U, AP and AtP are increased, but SV is slightly decreased (Figure 4(a)). Note also that AtP max and AtP min are increased to a different extent; therefore, AtP pulse pressure is decreased. CO is transiently strongly decreased, but within about 20 s it recovers; in steady state it is slightly below the initial conditions level 4500 mL/min. At the next step, at 120 s, as afterload is increased from 7 U to 10 U, a similar further increase of AP and AtP and a slight SV decrease occur. CO is further slightly decreased. AP, AtP, and VV return to initial conditions level. CO is transiently increased but within about 15 s it also returns to the initial condition level. The corresponding P-V loop diagram (Figure 4(b)) shows that, if preload is increased, both ESVV and EDVV are increased, but the latter to a smaller extent. Therefore, SV is slightly decreased (cf. Figure 4(a)).

The time course of cardiovascular variables during a single systole and diastole is presented in Figure 5, in columns A, B, and C, subdivided in four blocks. Upper block: pressures (AP, VP, and AtP) and flow (CO); middle two blocks: atrial and ventricular pressure at an enlarged pressure scale, and aortic and mitral flow; bottom block: ventricular volume (EDVV, ESVV, and SV).

The initial conditions, preload 6 mm Hg and afterload 4 U are shown in Figure 5(a). The time window is 57.6 s to 58.4 s. Peak VP is about 75 mm Hg. Aortic flow coincides with increase of VP-curve. dp/dt is 1886 mm Hg/s; TtP is 72 ms. During ICT and IRT there is no flow. The “effective” diastole starts as soon as VP is lower than AtP, thus inducing mitral flow. It has two peaks: a large early mitral flow in early diastole and a small late mitral flow in late diastole. The latter coincides with an increase in EDVV by about 20 mL. During IRT ventricle volume is constant, at ESVV. EDVV is about 118 mL, ESVV about 40 mL, and SV about 78 mL. End-systolic AtP is 4.5 mm Hg.

Figure 5(b) shows cardiovascular variables at maximum preload (i.e., increased by 33%, from 6 mm Hg to 8 mm Hg; time window is 177.6 s to 178.4). Peak VP is about 115 mm Hg. Aortic flow coincides with increase of VP-curve. The rate of ventricular contraction is strongly increased; dp/dt is increased from the resting value 1886 mm Hg/s to 3125 mm Hg/s; consequently, TtP is decreased from its resting value 72.0 ms to 63.6 ms. Peak VP coincides with peak aortic flow. Note that SV is increased to 115 mL mainly due an increased EDVV, about 158 mL, ESVV being virtually unchanged, about 40 mL. End-systolic AtP is 5.9 mm Hg.

Figure 5(c) shows cardiovascular variables at maximum afterload (i.e., increased by 250%, from 4 U to 10 U; time window is 177.6 s to 178.4). Peak VP is about 100 mm Hg and aortic flow coincides with the increase of VP-curve. The rate of ventricular contraction is mildly increased; dp/dt is slightly increased from the resting value 1886 mm Hg/s to 2127 mm Hg/s; consequently TtP is increased from its resting value 72.0 ms to 90.0 ms. Peak VP coincides with peak aortic flow. Note that EDVV is increased to about 128 mL and ESVV is also increased to about 60 mL. Therefore, SV is mildly decreased to 70 mL. End-systolic AtP is 4.5 mm Hg. The corresponding P-V loop diagram for Figure 5(c) is in Figure 5(d) which shows that, if preload is increased, the loop diagram becomes broader. It shows a high ventricular pressure, a high EDVV and SV. If afterload is increased, the loop diagram becomes narrower; ventricular pressure is increased. While ESVV and EDVV are increased to a different extent, SV is slightly decreased.

### 3.2. EEC Simulation of the Law of the Heart with Noncontracting Atrium

The effects of increasing preload or afterload on the time course of AP, CO, and AtP are very similar to those shown in Figure 5. The only difference is that CO is lower. Thus, in initial conditions CO is about 4200 mL/min. If atrium is contracting, in initial conditions CO is about 4500 mL/min.

The time course of cardiovascular variables during a single systole and diastole is presented in Figure 6: in initial conditions (Figure 6(a)), in maximum preload (Figure 6(b)), and in maximum afterload (Figure 6(c)). Note that—as atrium is not contracting—in the time course of AtP and of ventricular volume there is no “hump” in late diastole. Also, late mitral flow is absent. However, if atrium is not contracting, early mitral flow is very much pronounced; its peak value is larger (about 710 mL/s; Figure 6(a)) compared to its peak value when atrium is contracting (about 550 mL/s; Figure 5(a)).

The corresponding P-V loop diagram in Figure 6(d) is qualitatively almost the same as that shown in Figure 5(d). Quantitatively, however, the P-V loop diagram shows a smaller ventricle volume range and slightly lower ventricular pressure.

## 4. Discussion

### 4.1. General Comments

It should be noted that in the EEC, the heart valves have a negligible “back” flow. However, valve shunts (regurgitation) could be simulated, as has been shown recently [18]. Because blood inertia is not simulated, there is no dicrotic notch in pressure records. The pulse pressure is relatively large because the capacitance of the artificial aorta is relatively low. Therefore, the “Windkessel” effect is relatively small. Heart rate is constant (in the EEC it is 60/min) because in a Frank-Starling preparation there is no nervous or humoral influence. The discontinuities which seem to appear variables in Figures 5 and 6 are due to the fact that they are recorded for time span of 0.4 s and not 1 s (the complete heart cycle). If required, effects of heart rate and velocity of ventricular contraction could be studied as shown [17]. In the earlier simulation of the Starling preparation [16] as well as of the human circulation [17] the duration of systole was 0.25 s. After the circuitry was upgraded (including negative intrathoracic pressure and negative feedback) this value was decreased to 0.2 s and used in all simulations which followed.

When discussing the Starling heart-lung preparation it should be borne in mind that its behavior critically depends on the experimental layout; either the so-called “closed-circuit heart-lung preparation” [24] or the so-called called “open-circuit heart-lung preparation” [25] (also reviewed by [20, 21]).

In the “closed-circuit heart-lung preparation” blood volume is constant. The pumping action of the heart is determined not only by preload and afterload, but also by the capacitance of the attached artificial vascular circuit [16]. Its basic behavior is similar to the simulation of a simplified human cardiovascular system [16] which has been continuously upgraded [18, 19].

To demonstrate The Law of the Heart the so-called “open-circuit heart-lung preparation” (Figure 1(a)) should be used as already reported [16]. The attached “vascular” circuit should be very much simplified and include a venous reservoir [16]. It should be remembered, however, that also in this case vascular circuit is elastic. The blood volume within the circuit is not constant and depends on pressure in various sections. If for example, preload is increased, the “venous” volume and pressure are increased. As in the EEC, the blood flow meter is inserted at the end of the “venous” section and the increased preload transiently decreases CO. However, a new, higher steady-state level is established within about 10 s (Figure 3(a)). A similar, transient decrease in CO occurs if afterload is increased. However, as AP begins to increase, CO recovers gradually, in steady-state conditions CO is only slightly lower compared to initial conditions (Figure 4(a)). Changes in the time course of arterial pressure and ventricular volume agree well with the changes in the corresponding P-V loop diagrams (Figures 3(b) and 4(b)).

Simulations presented in this paper (e.g., aortic and ventricular pressure, ventricular volume) are qualitatively very similar to those described earlier ([16]; cf. [20, 21]). Quantitatively, however, there are many expected and readily explicable differences (cf. [25]).

### 4.2. Specific Comments on the Simulation of the Law of the Heart with Contracting Atrium

In the simulation of the Law of the Heart *quantitative* differences are expected because in the simulation circuit it is not possible to exactly match the size and detailed layout of the real heart-lung preparation. As shown in [25–27], the size of the mammalian ventricle (dogs and cats of variable weight) can vary considerably, resulting—even in the real heart-lung preparation—in a considerable variation of arterial pressure. *Qualitatively*, however, the presented simulations of the Law of the Heart agree very well with experimental data. For example, when preload was increased, in the animal heart-lung preparation, from 20 mm H_2_O to 210 mm H_2_O, cardiac output was increased from 40 mL/10 s to 250 mL/10 s [25]. Contrary to that, within wide limits, cardiac output was independent of arterial resistance (i.e., afterload [27]). If, for example, arterial resistance was increased from 44 mm Hg to 208 mm Hg, cardiac output remained constant at about 820 mL/min–860 mL/min as presented in [26] and reviewed in [20, 21]. In comparison, the corresponding results of the EEC computer simulation are as follows:

1. The stroke volume of the ventricle (and consequently cardiac output and AP pulse pressure) is highly increased if preload is increased from 6 mm Hg to 8 mm Hg (increased by a relatively small extent, by 33%). The ventricle is an input-sensitive pump (Figures 3–6).
2. The stroke volume of the ventricle (and consequently cardiac output and AP pulse pressure) is only slightly decreased if afterload is increased from 4 U to 10 U (increased considerably, by 250%). The ventricle is an *output-insensitive* pump (Figures 3–6).

To explain the basic findings above it should be remembered that, if atrium and ventricle are contracting/relaxing, blood circulates in the attached circuit. If so, atrial pressure is not exactly equal to preload. Atrial pressure is always slightly lower than preload (Figure 3). Due to blood flow, there is always a small pressure drop across the conduit between the mouth of the venous reservoir and atrium. In initial conditions, for example, when preload is 6 mm Hg, the AtP max/AtP min ratio is 5.2 mm Hg to 3.5 mm Hg (Figure 3). It should also be noted that, as a rule, the AtP pulse changes in parallel to the range of ventricular volume; the changes in EDVV, ESVV, and SV. If preload is increased from 6 mm Hg to 8 mm Hg (by 33%), both atrial pressure and its pulse pressure are increased; the AtP max/AtP min ratio is 6.7 mm Hg to 4.3 mm Hg (Figure 3(a)). End-systolic AtP is increased from 4.5 mm Hg to 5.9 mm Hg (Figures 5(a) and 5(b)). Similarly, SV is increased. Due to the increased ventricular filling (increased EDVV), the force and rate of ventricular contraction are increased and TtP is decreased. Therefore, CO, AP, and AP pulse pressure increase, and ICT remains virtually unchanged.

Afterload is determined predominantly by “peripheral” resistance; its large increase (250%) results in a relatively small decrease in cardiac output (−17%). Therefore, its effect can be neglected. If afterload is increased from 4 U to 10 U (by 250%), AtP is slightly increased while AtP pulse is decreased; the AtP max/AtP min ratio is 5.6 mm Hg to 3.9 mm Hg (Figure 4(a)). However, end-systolic AtP remains virtually unchanged, 4.5 mm Hg (Figures 5(a) and 5(c)). Therefore, EDVV is slightly increased, reflecting a slightly increased diastolic distension. Consequently, peak VP, peak AP are increased. dp/dt and TtP are mildly increased. This means a better filling of left ventricle (reflected by increased EDVV) but, on the other hand, a slightly decreased emptying of left ventricle (increased ESVV). All these changes almost fully compensate the increased afterload. Therefore SV, CO, and AP pulse pressure are only slightly decreased.

Somewhat unexpected is the finding that throughout early and mid diastole the atrial-ventricular pressure difference is very small. But despite this fact the early mitral flow is relatively large. This indicates that the ventricular filling is achieved mainly by its rapid relaxation. The late mitral flow in late diastole, affected by atrial contraction, is relatively large, but very brief, only about 0.1 s. Therefore, in resting conditions, atrial contraction contributes only about 20% to the increased end-diastolic volume of the ventricle; cardiac output is about 4570 mL/min.

### 4.3. Specific Comments on the Simulation of the Law of the Heart with Notcontracting Atrium

The effects of increasing preload or increasing afterload, when atrium is not contracting, are qualitatively the same as when atrium is contracting (Figure 6). Quantitatively, however, there are differences.

1. If atrium is not contracting, cardiac output is about 4200 mL/min, which is slightly lower compared to 4570 mL/min if atrium is contracting.
2. Late mitral flow, due to atrial contraction, is absent (Figure 6). However, the early mitral flow is larger compared to that if atrium is contracting. This finding is in agreement with that expressed above: the diastolic filling of the ventricle is achieved mainly through vigorous ventricular contraction followed by ventricle relaxation.
3. It should be noted that the early mitral flow commences as soon as atrial pressure equals the ventricular pressure, that is, when AtP = VP. If atrium is not contracting, this pressure equals 5.2 mm Hg (Figure 6(a)). Peak early mitral flow is about 710 mL/s. If atrium is contracting, this pressure is lower; it equals 4.5 mm Hg (Figure 5(a)). Peak early mitral flow is lower, about 550 mL/s (Figure 5(a)). Similar data are obtained by comparing Figures 6(b) (6.8 mm Hg) and 5(b) (5.9 mm Hg), or 6(c) (5.3 mm Hg) and 5(c) (4.5 mm Hg). This finding, although seemingly paradox, is in excellent agreement with the fact that the atrial pressure in late ventricular systole is the higher, the weaker is atrial contraction, and the lower is the cardiac output.

To summarize, if atrium is contracting, cardiac output is about 4500 mL/min and AtP, at the end of ventricular systole, is 4.5 mm Hg. Therefore, maximal early mitral flow is about 550 mL/s and is determined almost exclusively by AtP. If, however, atrium is not contracting, cardiac output is lower, about 4200 mL/min and AtP, at the end of ventricular systole, is higher, 5.2 mm Hg. Therefore, maximal early mitral flow is also higher, about 710 mL/s, and is determined almost exclusively by AtP. The increased early mitral flow is a partial compensation for the decreased total mitral flow, due to the absence of atrial contraction.

### 4.4. Answers to the Three Basic Questions on the Mechanical Properties of the Ventricle

**(1) What is the Role of Atrium in the Filling of the Ventricle, if the Latter is Subjected to Increased Preload or Increased Afterload?**

In resting conditions, the role of atrium in the filling of the ventricle is relatively small since firstly, the absence of atrial contraction is partially compensated with an increased early mitral flow and secondly, the ventricular filling is achieved mainly by its rapid relaxation. If the ventricle is subjected to increased preload or increased afterload, the rapid ventricular diastolic relaxation is the main mechanism for the ventricular diastolic filling.

**(2) Why is the Ventricle a Highly Input Sensitive Pump, but at the Same Time a Relatively Output Insensitive Pump?**

The ventricle is a highly input sensitive pump because the input pressure determines the diastolic distension and, consequently, the force of ventricular systolic contraction. On the other hand, the ventricle is a relatively output insensitive pump. Due to mechanisms described above, increased afterload results in an increased ventricular diastolic distension. Therefore, a more vigorous ventricular systolic contraction follows, almost compensating increased aortic pressure. Therefore, in resting conditions, ventricular (cardiac) output is almost independent of afterload.

**(3) What is the Change in the P-V Loop Diagram if the Ventricle is Subjected to Increased Preload or Increased Afterload?**

In increased preload the P-V loop diagram becomes broader (i.e., a shift to higher EDV with almost no change in ESV, which strongly increases SV) and higher (with higher ventricular pressure). In increased afterload the P-V loop diagram becomes narrower (i.e., both EDV and ESV are increased, but the former to a larger extent than the latter, which slightly decreases the SV) and higher.

## 5. Conclusions

To our knowledge, the presented EEC is the first computer simulation to explicitly simulate the Frank-Starling Law of the Heart. With the presented software, the Frank-Starling Law of the Heart can be demonstrated without the need to perform a technically difficult animal preparation that requires skilled staff and access to a suitable live animal model. Also, simulated measurements of selected cardiovascular variables (i.e., mitral or aortic flow) are possible and do not increase the cost or the complexity of the demonstration. Finally, when live animal preparations are impractical, the presented computer-based simulation offers a technically simple and low-cost alternative for demonstrating the Frank-Starling Law of the Heart. The presented EEC could be an excellent tool in cardiovascular research, on the one hand, to study how the ventricle, during contraction, adapts itself to a resistive or capacitive load, for example, in hypertension or in greatly changed aortic elastance. On the other hand, the ventricular relaxation, the ventricular filling process, and its dependence on the heart rate can be quantitatively studied in great detail.

## Figures and Tables

**Table 1:** Equivalent quantities and arbitrary units in simulation of cardiovascular system.

Electronic circuit	Unit(s)	Cardiovascular system	Unit(s)
Voltage	1 V	Pressure	10 mm Hg
Ground potential (reference for voltage measurements)	0 V	Atmospheric pressure (reference for pressure measurement)	0 mm Hg
Current	1 *μ*A = 60 *μ*As/min	Blood flow	100 mL/s = 6000 mL/min
Resistance	10 V/1 *μ*A = 10 MΩ	Resistance to flow	100 mm Hg/100 mL/s = 1 U
Capacitance	1 *μ*F = 1 *μ*As/1 V	Capacitance	100 mL/10 mm Hg
Charge	1 *μ*As	Volume	100 mL
Time	1 s	Time	1 s

**Table 2:** The recorded variables and their acronyms.

Aortic pressure	Ap
Atrial pressure	AtP
Maximal atrial pressure	ATP max
Minimal atrial pressure	ATP min
Cardiac output	CO
Ejection fraction of the ventricle	EF
End-diastolic volume	EDVV
End-systolic volume	ESVV
Isovolumic contraction time	ICT
Isovolumic relaxation time	IRT
Maximum velocity of ventricular contraction	dp/dt
Stroke volume	SV
Ventricular pressure	VP
Ventricle volume	VV
Time to peak (time from beginning of systole to peak ventricular pressure)	TtP
